# Supplementary material for: Interhospital Spread of blaVIM-1- and blaCTX-M-15-Producing K. pneumoniae ST15 on an IncR Plasmid in Southern Spain
Source: Antibiotics (Basel). 2023 Dec 13;12(12):1727. doi: 10.3390/antibiotics12121727 (PMC10740488; doi:10.3390/antibiotics12121727)
Supplement: Supplementary file 1 [file antibiotics-12-01727-s001.zip › Supplementary Table S1.pdf]

Supplementary Table S1. Antimicrobial resistance determinants of *K. pneumoniae* ST15 producing *bla*$_{VIM-1}$ and *bla*$_{CTX-M-15}$

	Antimicrobial resistance determinants			
Isolate (nº)	***B*-lactams**	**Quinolones**	**Aminoglycosides**	**Folate antagonist**
2017787	VIM-1/CTX-M-15/SHV-28/TEM-1/OXA-1	qnrB1/qnrB2	aph(3')-Ib/aph(6')-Id/ aac(3)-IIa/aadA1/aadA2	sul1/sul2/ dfrA12
20180256	VIM-1/CTX-M-15/SHV-28/TEM-1/OXA-1	qnrB1/qnrB2	aac(6')-Ib-cr/ aph(3')-Ib/aph(6')-Id/ aac(3)-IIa/aadA1/aadA2	sul1/sul2/ dfrA12
20180912	VIM-1/CTX-M-15/SHV-28/TEM-1/OXA-1	qnrB2	aac(6')-Ib-cr/ aph(3')-Ib/aph(6')-Id/ aac(3)-IIa/aadA1	sul1/sul2
20181130	VIM-1/CTX-M-15/SHV-28/TEM-1/OXA-1	qnrB1/qnrB2	aac(6')-Ib-cr/ aph(3')-Ib/aph(6')-Id/ aac(3)-IIa/aadA1/aadA2	sul1/sul2/ dfrA12
20190090	VIM-1/CTX-M-15/SHV-28/TEM-1/OXA-1	qnrB1/qnrB2	aph(3')-Ib/aph(6')-Id/ aac(3)-IIa/aadA1/aadA2	sul1/sul2/ dfrA12
20190095	VIM-1/CTX-M-15/SHV-28/TEM-1/OXA-1	qnrB1/qnrB2	aac(6')-Ib-cr/ aph(3')-Ib/aph(6')-Id/ aac(3)-IIa/aadA1/aadA2	sul1/sul2/ dfrA12
20190191	VIM-1/CTX-M-15/SHV-28/TEM-1/OXA-1	qnrB1/qnrB2	aac(6')-Ib-cr/ aph(3')-Ib/aph(6')-Id/ aac(3)-IIa/aadA1/aadA2	sul1/sul2/ dfrA12
