# Supplementary material for: Interhospital Spread of blaVIM-1- and blaCTX-M-15-Producing K. pneumoniae ST15 on an IncR Plasmid in Southern Spain
Source: Antibiotics (Basel). 2023 Dec 13;12(12):1727. doi: 10.3390/antibiotics12121727 (PMC10740488; doi:10.3390/antibiotics12121727)
Supplement: Supplementary file 1 [file antibiotics-12-01727-s001.zip › Supplementary Figure S1.pdf]

**Supplementary Figure S1**: Clonal relatedness of *XbaI*-digested DNA by pulsed-field gel electrophoresis analysis (PFGE).

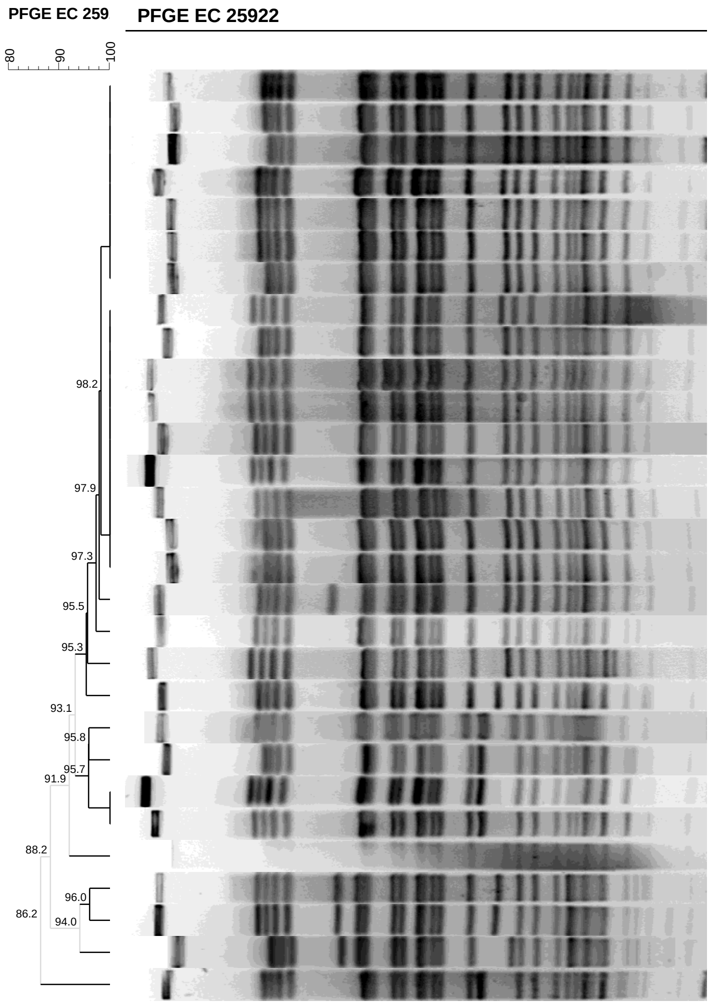


| | Isolate nº | *bla* | Date | Hospital | Clon | cgMLST |
|---|---|---|---|---|---|---|
| | 20190096 | VIM-1+CTX-M-15+SHV-28+TEM-1 | 13/02/19 | H U Virgen Macarena | ST15 | |
| | 20190717 | VIM-1+CTX-M-15+SHV-28+TEM-1 | 14/10/19 | H U Virgen Macarena | ST15 | |
| WGS | 20190191 | VIM-1+CTX-M-15+SHV-28+TEM-1 | 22/02/19 | H Juan Ramón JImé. | ST15 | 3266 |
| | 20190286 | VIM-1+CTX-M-15+SHV-28+TEM-1 | 12/05/19 | H U Virgen Macarena | ST15 | |
| | 20190402 | VIM-1+CTX-M-15+SHV-28+TEM-1 | 01/07/19 | H U Virgen Macarena | ST15 | |
| | 20190403 | VIM-1+CTX-M-15+SHV-28+TEM-1 | 01/07/19 | H U Virgen Macarena | ST15 | |
| | 20190526 | VIM-1+CTX-M-15+SHV-28+TEM-1 | 14/08/19 | H U Virgen Macarena | ST15 | |
| WGS | 2017787 | VIM-1+CTX-M-15+SHV-28+TEM-1 | 14/08/17 | H Infanta Elena | ST15 | 3271 |
| | 2017917 | VIM-1+CTX-M-15+SHV-28+TEM-1 | 15/09/17 | H Infanta Elena | ST15 | |
| | 20181086 | VIM-1+CTX-M-15+SHV-28+TEM-1 | 29/11/18 | H Infanta Elena | ST15 | |
| | 20181087 | VIM-1+CTX-M-15+SHV-28 | 30/11/18 | H Infanta Elena | ST15 | |
| WGS | 20181130 | VIM-1+CTX-M-15+SHV-28+TEM-1 | 13/12/18 | H U Virgen Macarena | ST15 | 3266 |
| | 20190260 | VIM-1+CTX-M-15+SHV-28+TEM-1 | 02/05/19 | H U Virgen Macarena | ST15 | |
| | 20190504 | VIM-1+CTX-M-15+SHV-28+TEM-1 | 24/07/19 | H U Virgen Macarena | ST15 | |
| | 20190523 | VIM-1+CTX-M-15+SHV-28+TEM-1 | 14/08/19 | H U Virgen Macarena | ST15 | |
| | 20190524 | VIM-1+CTX-M-15+SHV-28+TEM-1 | 14/08/19 | H U Virgen Macarena | ST15 | |
| | 20190175 | VIM-1+CTX-M-15+SHV-28+TEM-1 | 13/03/19 | H U Virgen Macarena | ST15 | |
| | 20190303 | VIM-1+CTX-M-15+SHV-28+TEM-1 | 17/05/19 | H U Virgen Macarena | ST15 | |
| WGS | 20180912 | VIM-1+CTX-M-15+SHV-28 | 05/10/18 | H Infanta Elena | ST15 | 3266 |
| | 20180784 | VIM-1+CTX-M-15+SHV-28+TEM-1 | 05/09/18 | H Infanta Elena | ST15 | |
| | 20180797 | VIM-1+CTX-M-15+SHV-28+TEM-1 | 15/09/18 | H Infanta Elena | ST15 | |
| WGS | 20190090 | VIM-1+CTX-M-15+SHV-28+TEM-1 | 31/01/19 | H Juan Ramón Jimé. | ST15 | 3266 |
| | 20190268 | VIM-1+CTX-M-15+SHV-28+TEM-1 | 07/05/19 | H U Virgen Macarena | ST15 | |
| | 20190693 | VIM-1+CTX-M-15+SHV-28+TEM-1 | 24/09/19 | H de la Merced | ST15 | |
| | 20190944 | VIM-1+CTX-M-15+SHV-28+TEM-1 | 20/12/19 | H U Virgen Macarena | ST15 | |
| WGS | 20180256 | VIM-1+CTX-M-15+SHV-28+TEM-1 | 16/03/18 | H U Virgen Macarena | ST15 | 3266 |
| | 20180516 | VIM-1+CTX-M-15+SHV-28+TEM-1 | 24/05/18 | H Infanta Elena | ST15 | |
| | 20190643 | VIM-1+CTX-M-15+SHV-28 | 05/09/19 | H Infanta Elena | ST15 | 5114 |
| WGS | 20190095 | VIM-1+CTX-M-15+SHV-28+TEM-1 | 07/02/19 | H Juan Ramón Jimé. | ST15 | 4847 |
