# Supplementary material for: Interhospital Spread of blaVIM-1- and blaCTX-M-15-Producing K. pneumoniae ST15 on an IncR Plasmid in Southern Spain
Source: Antibiotics (Basel). 2023 Dec 13;12(12):1727. doi: 10.3390/antibiotics12121727 (PMC10740488; doi:10.3390/antibiotics12121727)
Supplement: Supplementary file 1 [file antibiotics-12-01727-s001.zip › Supplementary Figure S2.pdf]

Supplementary Figure S2: Matrix of 1133 out of 4891 alleles; not taking into account missing values of *K. pneumoniae* ST15 producing *bla*$_{VIM-1}$ and *bla*$_{CTX-M-15}$, the 13 contemporary genomes of ST15 isolates from other centers in the region and the 2 genomes of VIM-1-producing ST15 from Spanish regions other than Andalusia.

Center		2017787	20190090	20190912	20190191	20181130	20190095	20180256	20190368	2017982	EuSCAPE_ES133	20180793	EuSCAPE_ES080	20180821	20190819	20180679	20190675	2017414	20190545	20190784	20190653	20190943	20180964
H Infanta Elena	2017787	0	0	2	2	5	6	27	165	167	171	172	174	175	177	178	180	182	183	197	210	210	226
H Juan Ramón Jiménez	20190090	0	0	2	2	5	6	27	165	167	171	172	174	175	177	178	180	182	182	197	210	210	225
H Infanta Elena	20190912	2	2	0	4	8	8	28	161	163	168	168	170	171	173	172	176	179	178	194	177	176	194
H Juan Ramón Jiménez	20190191	2	2	4	0	8	8	29	165	167	172	172	175	175	178	177	181	183	183	196	211	211	226
H Virgen Macarena	20181130	5	5	8	8	0	11	31	169	170	175	176	178	179	179	182	182	186	186	201	214	213	228
H Juan Ramón Jiménez	20190095_	6	6	8	8	11	0	33	169	171	177	176	179	179	181	183	184	187	187	199	215	215	229
H Virgen Macarena	20180256	27	27	28	29	31	33	0	144	146	152	151	155	154	156	159	158	161	162	176	187	187	204
	20190368	165	165	161	165	169	169	144	0	29	147	33	149	36	40	156	163	163	163	182	203	203	209
	2017982	167	167	163	167	170	171	146	29	0	150	24	151	27	12	156	163	163	164	183	205	205	214
	EuSCAPE_	171	171	168	172	175	177	152	147	150	0	153	27	156	160	151	152	157	155	174	206	207	210
	20180793	172	172	168	172	176	176	151	33	24	153	0	154	5	36	161	167	167	167	187	209	210	217
	EuSCAPE_	174	174	170	175	178	179	155	149	151	27	154	0	157	160	151	154	160	156	175	209	209	212
	20180821	175	175	171	175	179	179	154	36	27	156	5	157	0	39	163	170	170	170	189	213	213	220
	20190819	177	177	173	178	179	181	156	40	12	160	36	160	39	0	166	172	173	172	194	216	215	225
	20180679	178	178	172	177	182	183	159	156	156	151	161	151	163	166	0	109	112	112	133	197	198	190
	20190675	180	180	176	181	182	184	158	163	163	152	167	154	170	172	109	0	45	10	138	210	210	203
	2017414	182	182	179	183	186	187	161	163	163	157	167	160	170	173	112	45	0	51	141	209	210	205
	20190545	183	182	178	183	186	187	162	163	164	155	167	156	170	172	112	10	51	0	144	214	214	205
	20190784	197	197	194	196	201	199	176	182	183	174	187	175	189	194	133	138	141	144	0	233	233	221
	20190653	210	210	177	211	214	215	187	203	205	206	209	209	213	216	197	210	209	214	233	0	62	200
	20190943	210	210	176	211	213	215	187	203	205	207	210	209	213	215	198	210	210	214	233	62	0	202
	20180964	226	225	194	226	228	229	204	209	214	210	217	212	220	225	190	203	205	205	221	200	202	0

0 alleles	
1-10 alleles	
11-20 alleles	
21-50 alleles	
>50 alleles	
